# Tumorigenicity and prediction of clinical prognosis of patient‐derived gastric cancer organoids

**DOI:** 10.1002/ctm2.1588

**Published:** 2024-02-16

**Authors:** Ting Wang, Wanlu Song, Qingyu Meng, Chuanqing Qu, Shaohua Guo, Yalong Wang, Ronghui Tan, Baoqing Jia, Ye‐Guang Chen

**Affiliations:** ^1^ The State Key Laboratory of Membrane Biology Tsinghua‐Peking Center for Life Sciences School of Life Sciences Tsinghua University Beijing China; ^2^ Department of General Surgery The First Medical Center PLA General Hospital Beijing China; ^3^ Guangzhou National Laboratory Guangzhou China; ^4^ School of Basic Medicine Jiangxi Medical College Nanchang University Nanchang China

Dear Editor,

1

In this study, we established a biobank of gastric tumour organoids (GTOs), which retained primary tumour characteristics. The response of GTOs to drugs was correlated with individual patient's long‐term postoperative prognosis, suggesting that GTOs are a good model for drug prediction for gastric cancer (GC) treatment.

Varying response to chemotherapy restricts GC treatment efficacy and clinical prognosis.^1^ GC‐derived organoids are a good model for drug tests as they preserve primary tumour features.^2^, ^3^, ^4^, ^5^ However, the correlation between patients' long‐term clinical prognosis and drug sensitivity is unclear. The distinction between GTOs and gastric normal organoids (GNOs) is vague. To address these questions, we established GTOs and corresponding GNOs from 17 GC patients: seven intestinal‐type, eight diffuse‐type and two mixed‐type (Table S1).^6^ Two types of GTO morphology were observed: one displaying a glandular, round pattern with an obvious lumen, similar to intestinal‐type tumours; the other exhibiting a solid morphology with the nucleus shifting to one side and resembling diffuse‐type tumours (Figure 1A,B). GTOs derived from mixed‐type GC tissues displayed both types of morphology (Figure 1C). All GNOs derived from normal tissues displayed glandular structures, mimicking normal gastric glands (Figure 1D).

**FIGURE 1:**
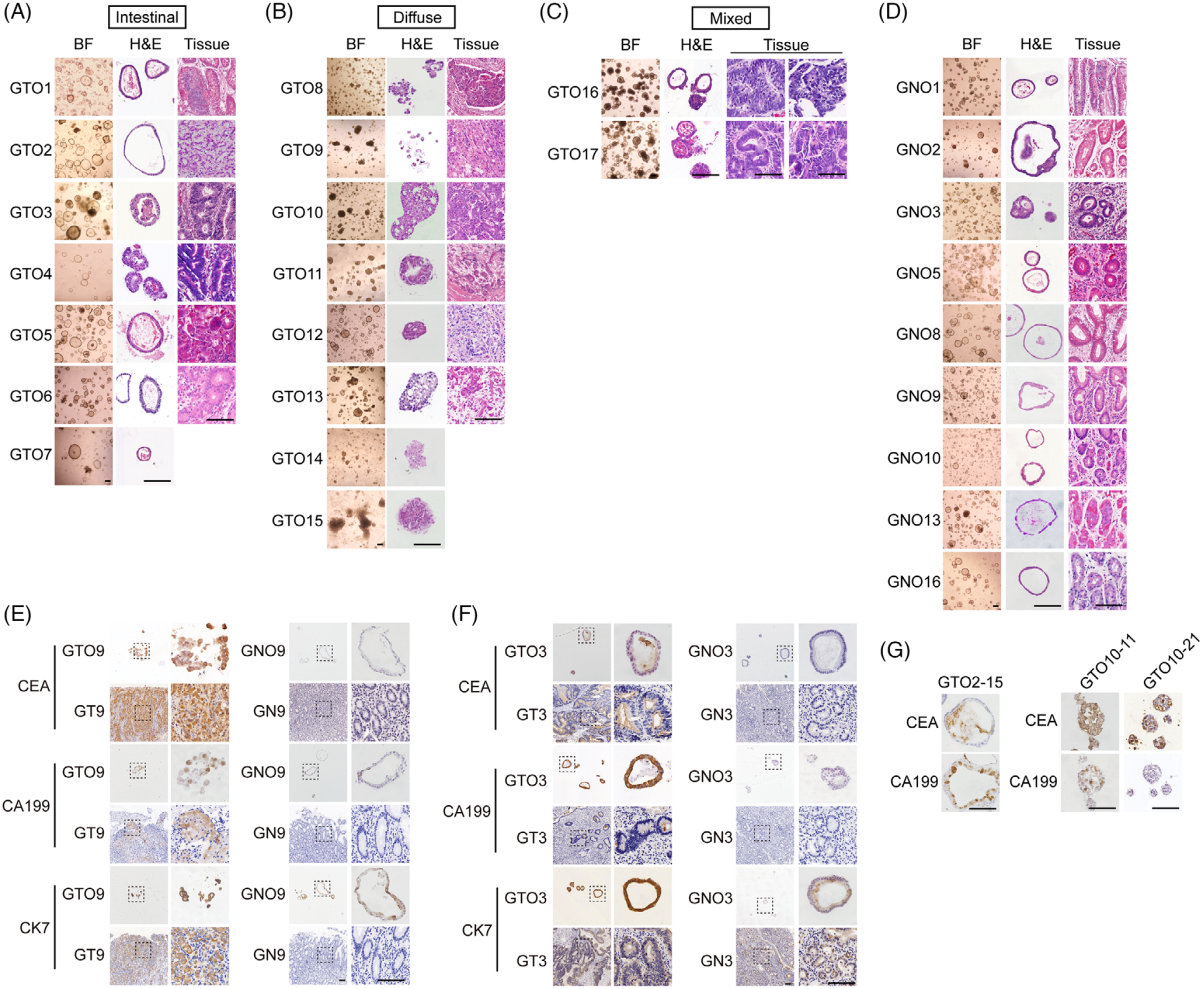
Gastric tumour organoids (GTOs) maintain the histological characteristics of primary tumour tissues. (A–C) Representative microscopy images and Hematoxylin and Eosin (H&E) staining of intestinal‐type GTOs (A), diffuse‐type GTOs (B) mixed‐type GTOs (C) and primary tumour tissues. (D) Representative microscopy images and H&E staining of gastric normal organoids (GNOs), along with primary normal tissues. Scale bars in the brightfield images are 200 μm, while scale bars in H&E images for organoids and paired tissues are 100 μm (A–D). (E, F) Immunohistochemical staining of tumour markers carcinoembryonic antigen (CEA), carbohydrate antigen 19‐9 (CA19‐9) and cytokeratin 7 (CK7) in GTOs, GNOs, and relevant tumour and normal tissues. Scale bars: 100 μm. (G) Immunohistochemical staining of CEA and CA19‐9 in long‐term passages of GTO2 (GTO2‐15) and GTO10 (GTO1011 and GTO1021). Scale bars: 100 μm. GT: gastric tumour tissue; GN: gastric normal tissue; GTO: gastric tumour organoid; GNO: gastric normal organoid. The three tissues missing are the lack of paired tumour tissues, which were biopsy samples (A, B).

To assess GTOs’ tumour features, gastric tumour markers carcinoembryonic antigen (CEA), carbohydrate antigen 19‐9 (CA19‐9), and cytokeratin 7 (CK7) were examined. CEA and CA19‐9 expression were detected in GTO2, GTO3, GTO9, GTO13, GTO2‐15, GTO10‐11 and GTO10‐21 and their corresponding GTs, but not in GNOs and normal tissues (Figure 1E–G and S1A‐C). CEA and CA19‐9 expression were found in 13 GTOs and their corresponding GTs (11/13 and 12/13, respectively) (Table S2). Therefore, GTOs preserved tumour marker expression of tumours, even in long‐term cultures.

Whole exome sequencing revealed analogous tumour mutational burden in GTOs (Figure S2A and Table S3). Furthermore, gene mutations were correlated with specific clinical features (Figure S2B‐D and Table S4). Single nucleotide variants for different passages of GTO9 and GTO10 exhibited significant similarity to primary tumours (Figure S3A,B). While gene mutations in GT9 were retained in long‐term passages of GTO9, some GTOs acquired additional mutations during prolonged culture (GTO12‐22 and GTO17‐22) (Figure 2A), possibly due to growth competition among different subclones.^7^ The base substitutions exhibited notable similarities in most matched organoids and GTs (Figure 2B). A substantial proportion of mutations were common mutations (Figure 2C,D). Mutational signatures were also similar between GTOs and GTs (Figure 2E, S3C and Table S6). These findings suggest that GTOs effectively preserve mutation characteristics of primary tissues.

**FIGURE 2:**
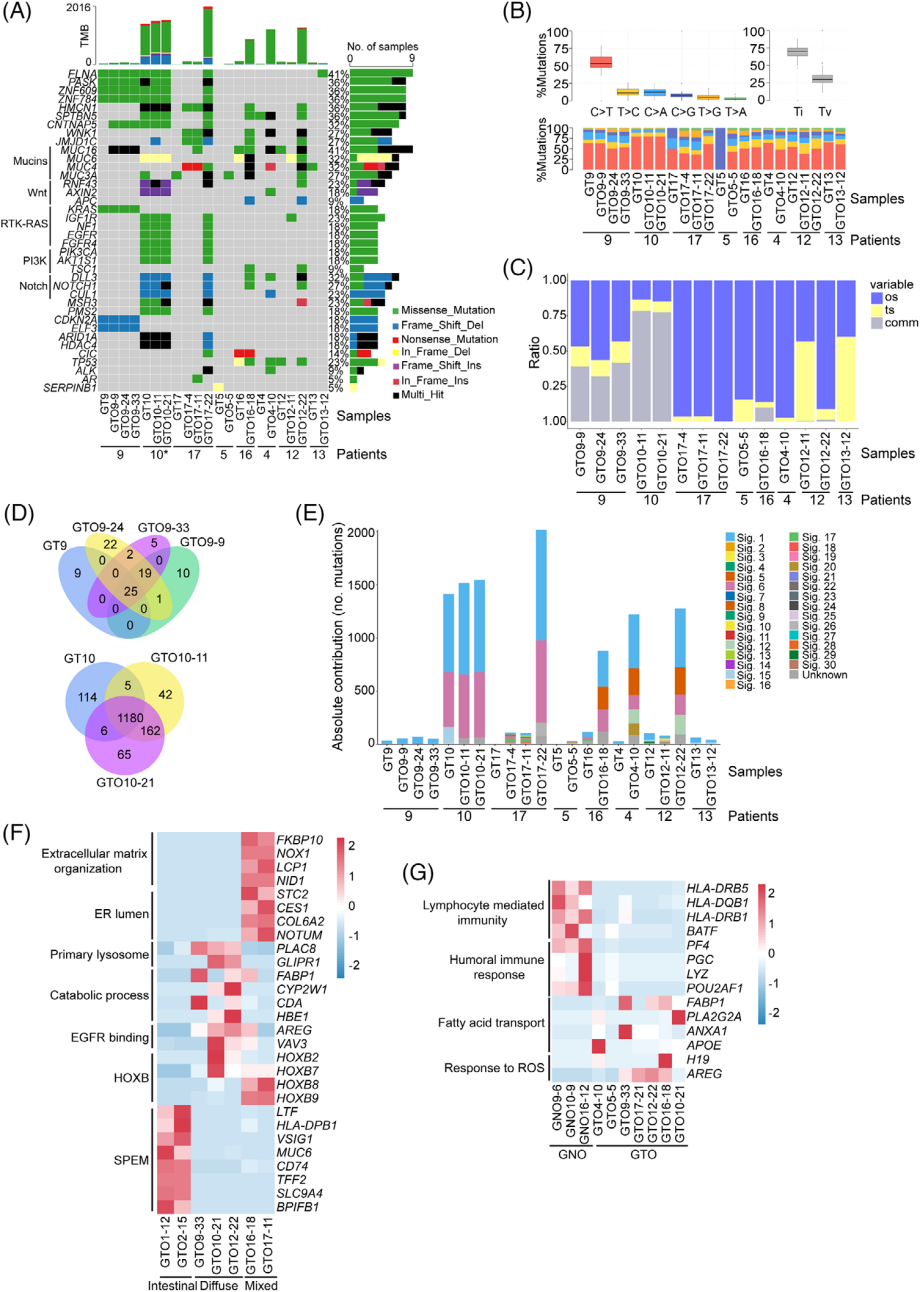
Gastric tumour organoids (GTOs) preserve the genetic characteristics of primary tumor tissues. (A) Top mutated genes and molecular drivers of gastric cancer in GTOs at different passages, along with primary gastric tumour (GT) tissues. The asterisk indicates MSI‐H status in the organoids (GTO10s) and tumour tissue (GT10) of patient 10. The patient numbers are represented below the sample names. (B) Base mutation patterns in GTOs of different passages and primary tumor tissues. The upper panel boxplot presents an overview of the distribution patterns of six distinct conversion types, encompassing transitions and transversions. The lower panel barplot showcases the relative proportion of conversions within each sample. The patient numbers are represented below the sample names. Ti, transition; Tv, transversion. (C) Mutational accordance between GTOs of different passages and primary tumor tissues. The patient numbers are represented below the sample names. Os, organoid‐specific mutations; ts, tissue‐specific mutations; comm, common mutations shared among primary tumour tissues and tumour organoids. (D) Venn diagrams depicting the mutational accordance between GTOs of different passages and primary tumor tissues. In GTO9s, a substantial proportion of mutations were observed to be common mutations, with approximately 30% of mutations shared among GT9 and GTO9s. The Venn diagram revealed a 73% overlap in the mutational spectrum among GTO9s and their parental tissue (C, D). In patient 10, GTO10s shared 70% of mutations with the tissue, and there was 87% concurrence among GTO10s and GT10 shown in the Venn diagram (C, D). (E) Absolute contribution of mutational signatures across GTOs and paired tumor tissues. Different colours represent 31 kinds of signatures. The patient numbers are represented below the sample names. Sig: signature. (F) Heatmap of differentially expressed genes among intestinal‐type, diffuse‐type and mixed‐type GTOs based on the Lauren classification. In detail, mixed‐type organoids exhibited upregulation of genes involved in extracellular matrix organization and endoplasmic reticulum (ER) lumen, which are related to tumour malignancy. Diffuse‐type organoids showed increased lysosome activity and catabolic processes, which are characteristics of tumor cells. Both diffuse‐type and mixed‐type organoids showed an upregulation of Homeobox B (HOXB) family genes, known to promote cancer progression. On the other hand, intestinal‐type organoids exhibited upregulation of genes related to spasmolytic polypeptide‐expressing metaplasia (SPEM), which aligns with pathological features of intestinal‐type gastric cancer. (G) Heatmap of differentially expressed genes in gastric normal organoids (GNOs) compared to GTOs. GT: gastric tumour tissue; GN: gastric normal tissue; GTO: gastric tumour organoid; GNO: gastric normal organoid. The comparison between GNOs and GTOs revealed downregulation of MHC class II‐associated lymphocyte immunity and humoral immune response in GTOs. Conversely, fatty acid transport and the genes involved in reactive oxygen species (ROS) were upregulated in GTOs, closely associated with tumorigenesis.

Gene expression profiling revealed a robust correlation between GTOs and GTs (Figure S4A and Table S7). Principal Components Analysis showed a close proximity within GTO subtypes (Figure S4B,C). Compared to GT, GTOs had high expression in genes linked to xenobiotic stimuli and low in the ones associated with immune response and fibroblasts (Figure S4D). Distinct gene expression profiles were also observed in three types of organoids (Figure 2F). Compared to GNOs, immune‐related genes (lymphocyte‐mediated immunity and humoral immune response) were downregulated in GTOs (Figure 2G).

To evaluate GTO tumorigenicity, we conducted GTO xenograft (GTOX) experiments. Most diffuse‐type and mixed‐type GTOs displayed strong tumorigenicity, and the GTOXs exhibited similar structures to the original GTs and were CEA^+^ (Figure 3). However, some GTOs had low or no tumorigenicity, and the GTOXs displayed connective tissue‐like neoplasms (Figure 3). GNOs only formed cysts (Figure S5A,B). Variations in GTO tumorigenicity are possibly linked to WNT mutations, which requires further exploration (Figure S5C).

**FIGURE 3:**
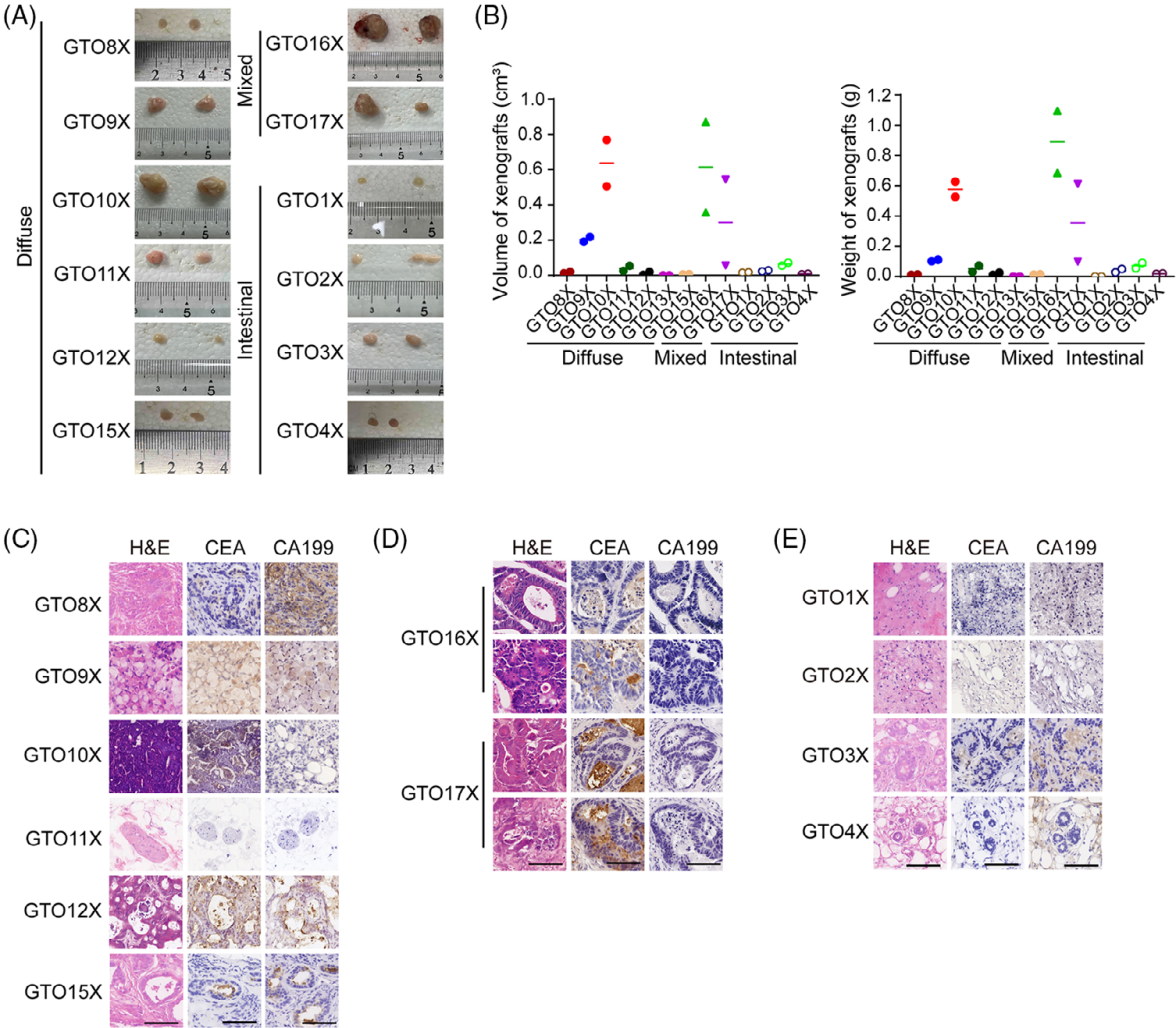
Gastric tumour organoids (GTOs) retain tumorigenesis. (A) Xenografts generated by GTOs in NCG‐immunodeficient mice. (B) Volume and weight of GTO xenografts. (C–E) Hematoxylin and Eosin (H&E) and immunohistochemical staining of carcinoembryonic antigen (CEA) and carbohydrate antigen 19‐9 (CA19‐9) in GTO xenografts. Scale bars: 100 μm.

To simulate the therapeutic effects of drugs, GTOs were treated with various drugs. The drug resistance of GTOs was correlated with late stages of GC but had no significant associations with gender, age, or tumour classification (Figure 4A and Table S8). GTO10 was more sensitive to oxaliplatin than to 5‑fluorouracil (5‑FU)/oxaliplatin (Figure 4A and Table S9), consistent with MSI‐H status^8^ (Table S5) and good outcome of oxaliplatin monotherapy in this patient (Figure S6A). Conversely, patients 4, 6 and 9 treated with 5‐FU/oxaliplatin had a poor outcome and exhibited resistance in GTOs. A significant correlation was also observed between progression‐free survival time and cell viability in GTOs (Figure S6B). However, some GTO responses did not align with clinical outcomes, possibly due to peritoneal metastasis hindering drug delivery (patient 3). GTO4, which highly expressed *TOP1MT*, was sensitive to irinotecan,^9^ indicating potential as a second‐line treatment (Figure 4B and S6C, D). GTO10, with *PIK3CA* mutation, showed resistance to erlotinib and gefitinib (Figure 4A,C), and GTO9 with *KRAS* mutation exhibited sensitivity to gefitinib.^10^ These data underscore GTOs’ role in assessing drug response and optimizing treatment strategies, along with gene expression and mutations. Furthermore, most drugs showed consistent responses in GTO2, GTO12 and GTO17 with over 10 passages apart (Figure 4D).

**FIGURE 4:**
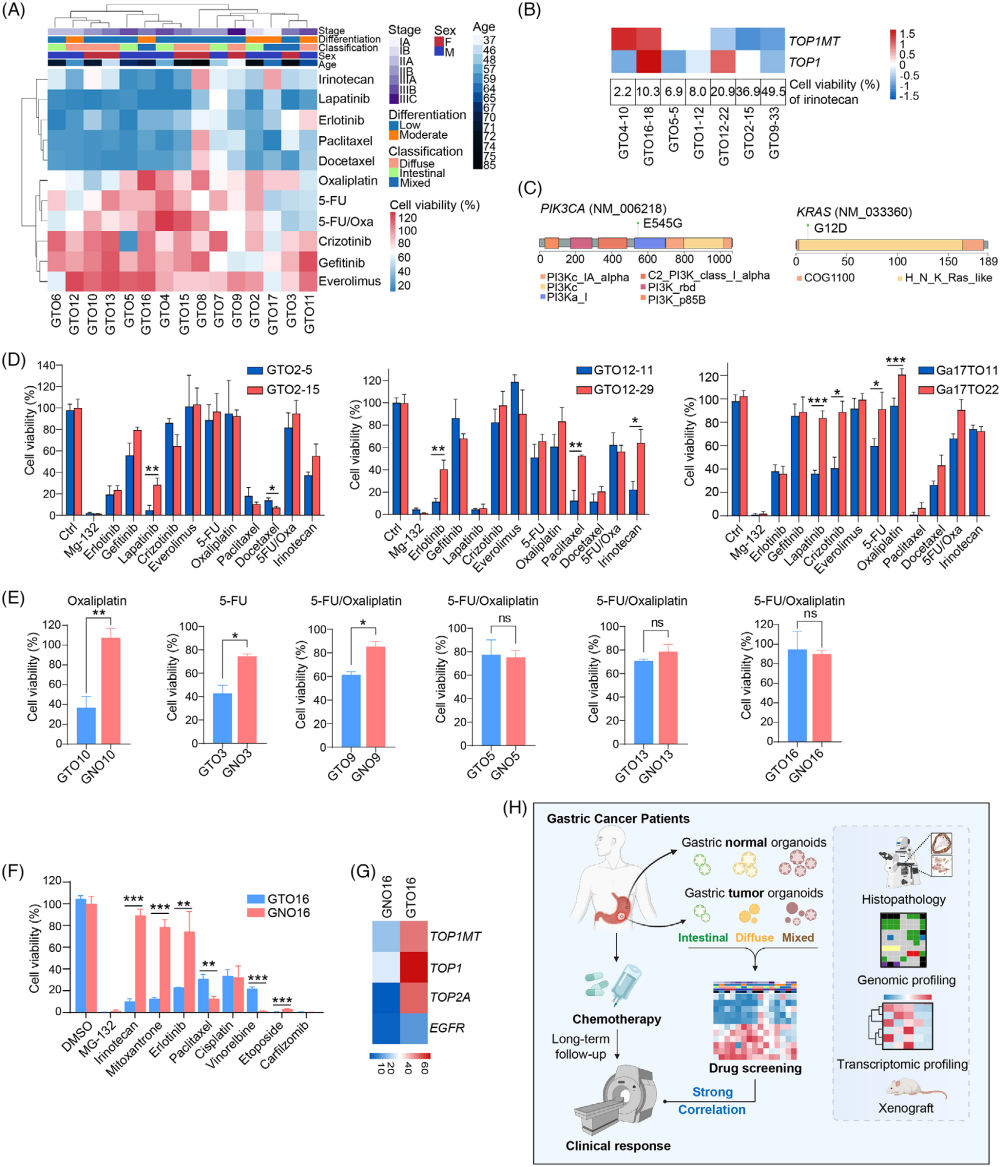
Gastric tumour organoids (GTOs) were employed in personalized medicine. (A) The responses of 15 GTO lines to various drug treatments were clustered according to the cell viability values versus the control group. The annotated information includes sex, age, degree of gastric cancer differentiation, Lauren's classification and AJCC stage. (B) Heatmap showing the Z‐score of *TOP1MT* and *TOP1* genes, and cell viability upon irinotecan treatment versus control group in GTOs. Irinotecan: a DNA topoisomerase inhibitor. (C) The amino acid missense mutations of *PIK3CA* and *KRAS* are represented in a lollipop plot by maftools package, with each domain labelled in different colours. Erlotinib and gefitinib: EGFR inhibitors. (D) Two passages of GTO2, GTO12 and GTO17 were treated with therapeutic drugs and the cell viability values were measured. (E) Responses of GTOs and gastric normal organoids (GNOs) from the same patient to different drug treatments versus the control group. (F) Responses of GTO16 and GNO16 to different drug treatments versus control group. Mitoxantrone: a topoisomerase II alpha (TOP2A) inhibitor. (G) Heatmap showing the Z‐score of *TOP1MT, TOP1, TOP2A* and *EGFR* genes in GTO16 and GNO16. Organoids treated with DMSO served as the vehicle control, while those treated with MG‐132 served as the positive control. The cell viability value = survival cell number after drug treatment divided by cell number treated with DMSO ×100%. Statistical analysis was performed by a two‐tailed Student's *t*‐test. * Indicates *p* < .05, ** indicates *p* < .01, *** indicates *p* < .001, ns indicates no significance. Oxa: oxaliplatin. (H) The schematic representation illustrates that a living biobank comprising three subtypes of GTOs and corresponding GNOs was established from gastric cancer (GC) patients and analyzed through multi‐omics analysis. Furthermore, the response of GTOs to drugs was correlated with clinical treatment outcomes in individual patients with long‐term follow‐up, suggesting that GTOs are a good model for drug prediction for GC treatment.

We then evaluated drug side effects using GNOs. Oxaliplatin exhibited low toxicity in GNO10 although displaying high efficacy to induce cell death in GTO10 (Figure 4E), consistent with patient 10's positive response to oxaliplatin. Similar results were observed in organoids of patients 3 and 9. Irinotecan, mitoxantrone and erlotinib demonstrated significant efficacy in GTO16 with minimal cytotoxicity in GNO16 (Figure 4F, S6E and Table S8). Likewise, mitoxantrone and erlotinib showed a similar pattern, consistent with gene expression (Figure 4F,G and S6E). Therefore, GNOs can be used for drug toxicity assessment.

In summary, our findings demonstrated that GTOs can faithfully recapture tumour features of original tumours and preserve genetic alterations after long‐term culture. We found distinct expression profiles among GTO subtypes, reflecting clinicopathological features of GC subtypes. Finally, we observed consistency between clinical prognosis and drug sensitivity in GTOs, indicating the potential use of GTOs for personalized medicine.

## AUTHOR CONTRIBUTIONS

Ting Wang and Ye‐Guang Chen conceived the study, analyzed the data and wrote the manuscript; Ting Wang and Chuanqing Qu performed the experiments; Wanlu Song performed bioinformatics analysis; Baoqing Jia, Qingyu Meng and Shaohua Guo provided specimens; Yalong Wang and Ronghui Tan revised the manuscript.

## CONFLICT OF INTEREST STATEMENT

The authors declare no conflict of interest.

## ETHICS STATEMENT

The approval number is 2019−346 from the PLA General Hospital Ethics Committee.

## Supporting information

Supplementary Materials and Figures

Table S1. Clinicopathological information for each patient

Table S2. Detailed levels of CEA and CA199 in GTOs and primary tumor tissues

Table S3. Mutations for all samples

Table S4. Critical gene mutations enriched within diverse clinicopathological patients

Table S5. MSI scores of all samples

Table S6. The composition of the signatures for each sample

Table S7. TPM values for all samples

Table S8. Steadystate plasma concentration of indicated therapeutic drugs

Table S9. Patients clinical outcomes and drug‐induced cell viability in GTOs

## Data Availability

The genomic data are in https://www.ncbi.nlm.nih.gov/geo/query/acc.cgi?acc=GSE235912.
