# Supplementary material for: Tumorigenicity and prediction of clinical prognosis of patient‐derived gastric cancer organoids
Source: Clin Transl Med. 2024 Feb 16;14(2):e1588. doi: 10.1002/ctm2.1588 (PMC10870796; doi:10.1002/ctm2.1588)
Supplement: Supplementary file 1 — Supplementary Materials and Figures [file CTM2-14-e1588-s005.docx]

**Supplementary information**

**Tumorigenicity and prediction of clinical prognosis chemotherapy response of patient-derived gastric cancer organoids**

Ting Wang^1*^, Wanlu Song^1*^, Qingyu Meng^2*^, Chuanqing Qu^1^, Shaohua Guo^2^, Yalong Wang^3^, Ronghui Tan^3^, Baoqing Jia^2#^, and Ye-Guang Chen^1,3,4#^

**Materials and Methods**

**Supplementary Figures S1-S6**

**Supplementary Tables S1-S9**

**Materials and methods**

**Human tissue collection and ethics statement**

Freshly processed human gastric tumor tissues and normal tissues located at least 5 cm away from the tumor border were collected from gastric cancer patients who underwent gastrectomy at the Department of General Surgery, The First Medical Center, PLA General Hospital in Beijing, China ^1^. The identification of normal tissues revealed no clear tumors, precancerous lesions, and benign diseases. The collection and use of these samples were conducted following the guidelines outlined in the Declaration of Helsinki, and the study protocol was approved by the PLA General Hospital Medical Science Research Ethics Committee (approval number: 2019-346).

**Gastric organoid culture**

Gastric normal organoids were generated as following. Initially, normal tissues were washed twice with HBSS containing 1x Penicillin/Streptomycin (P/S). The tissues were then dissected to remove the muscle and mucus using scissors. Subsequently, the normal tissues were incubated in a solution containing HBSS supplemented with 10 mM EDTA, 0.5 mM DTT, 1x P/S, and 1 mg/μl Primocin at 4°C for 45 minutes while being gently rotated on a rotator. Afterward, the tissues were transferred to a 10 cm dish, and the gastric glands were released by scraping the epithelial side using a glass slide. The released gastric glands were washed twice with HBSS, centrifuged, and suspended in Matrigel. A 40 μl mixture of the suspended glands and Matrigel was added to each well of a 24-well plate. Once the drops solidified, 500 μl of gastric organoid medium (advanced DMEM/F12, 1x GlutaMax, 1x HEPES, 1x P/S, 1x B27, CHIR-99021, R-spondin-1, Noggin, EGF, FGF10, N-Acetylcysteine, Gastrin, SB431542, Y-27632, and Primocin) was added to each well. Normal organoids typically appeared after 5-7 days and were passaged every 7-10 days to maintain their growth and viability. Eight out of 17 GNOs are not available to present in **Fig. 1D** because some tumor samples were biopsies, which lacked paired normal tissues during sample collecting.

For tumor organoids, the protocol was similar with some modifications. The solid tumor sample was first washed with advanced DMEM/F12 containing 1x Penicillin/Streptomycin (P/S). After removing the outer layer of the tumor mass, the remaining tissue was cut to release the glands, which were collected and plated on a 24-well plate to serve as the first organoids. The remaining debris was incubated in HBSS supplemented with 10 mM EDTA, 0.5 mM DTT, 1x P/S, and 1 mg/μl Primocin at 4°C for 60 minutes while being rotated on a rotator. The tissues were subsequently transferred to a 10 cm dish, and the gastric glands were released by pipetting up and down 10 times in advanced DMEM/F12. The released glands were then washed twice with HBSS, centrifuged, and suspended in Matrigel to form the second organoids. To support their growth, 500 μl of gastric organoid medium (advanced DMEM/F12, 1x GlutaMax, 1x HEPES, 1x P/S, 1x B27, Wnt3a, R-spondin-1, Noggin, EGF, FGF10, N-Acetylcysteine, Gastrin, A83-01, Y-27632, and Primocin) was added to each well. Tumor organoids typically appeared after 5-7 days and were passaged every 7-10 days to maintain their growth and viability.

**Histochemistry and Immunohistochemistry**

Samples were fixed with 4% paraformaldehyde at 4°C overnight. Afterwards, they were paraffin-embedded and sectioned into 5 μm slices. The sections were then deparaffinized and subjected to histological analysis through staining with H&E and IHC assays. For IHC, tissues and organoid sections were subjected to the following steps: de-paraffinization in isopropanol and dehydration using a graded alcohol series. Antigen retrieval was performed, followed by permeabilization in PBS with 0.2% Triton X-100 at 4°C for 20 minutes. Subsequently, the sections were placed in a blocking buffer (1% BSA/PBS) for 45 minutes at room temperature. Primary antibodies, including mouse anti-CEA (ZM-0062, Origene), mouse anti-CA19-9 (ZM-0021, Origene), and rabbit anti-CK7 (1:100, 17513-1-AP, Proteintech), were added and incubated overnight at 4°C. For detection, HRP-conjugated goat anti-mouse and goat anti-rabbit secondary antibodies (PV-6002 and PV-6001, Origene) were applied for 90-120 minutes at room temperature. Images of the stained sections were acquired using the Digital Pathological Section Scanner (KF-PRO-120, KFBIO).

**Cell viability assay**

To simulate the therapeutic effects of drugs in the human body, tumor and normal organoids were treated with candidate drugs at steady-state plasma concentrations **(Table S8)** ^2,3^. Organoids were trypsinized to obtain a small and homogeneous size, and then plated on a 384-well microplate at a density of 1,000 cells per well, embedded in 50% Matrigel. TrypLE™ (12604013, Thermo Fisher) or Accutase (07920, Stemcell) was used for cell dissociation. The cells were plated in triplicate. The next day, cancer drugs were added to the medium using the Echo 550 acoustic dispenser (Labcyte). On day 6, cell viability based on ATP was quantified using the CellTiter-Glo® 3D Cell Viability Assay (G9682, Promega). The data represented the mean of three parallel replicates. Organoids treated with DMSO served as the vehicle control, while those treated with MG-132 served as the positive control.

**Whole-exome sequencing**

Total DNA was extracted from tissues and cultured organoids using the TIANamp Genomic DNA Kit (DP304, TIANGEN). Subsequently, DNA-sequencing libraries were prepared using the NEB Next® Ultra™ DNA Library Prep Kit for Illumina (NEB, USA) following the manufacturer's instructions. The DNA libraries were then subjected to sequencing using the Illumina Novaseq 6000 platform, generating 150 bp paired-end reads. The processing of the Whole-exome sequencing (WES) data was followed the best practice guidelines recommended by the Genome Analysis Toolkit. Somatic mutations were detected by comparing the genetic profiles of each tumor sample from tumor tissue and organoids to their corresponding normal samples. Initially, the raw FASTQ data were aligned to the human genome reference (GRCh38) using the Burrows-Wheeler Aligner (BWA) algorithm with default parameters of BWA-MEM. The resulting alignment files were sorted based on genomic coordinates using the Samtools. The tumor tissue and organoids samples were compared to paired normal samples. Subsequently, duplicate reads were marked and removed using Picard, and local realignment around indels was performed to enhance variant calling accuracy. Base quality scores were recalibrated using BQSR (Base Quality Score Recalibration) to correct systematic errors. Finally, variant calling was conducted using Mutect, and the called variants were subsequently filtered based on criteria such as read depth, mapping quality, allele frequency, and functional impact and SNV annotation was performed using ANNOVAR. The maftools package in R was utilized for visualization and exploration of the variant data, generating plots such as oncoplots, rainfall plots and lollipop plots. The mutation burden was also calculated and compared to TCGA using tcgaCompare of maftools.

Additionally, deconstructSigs was employed for mutational signature analysis using COSMIC (Catalogue Of Somatic Mutations In Cancer) database as a reference ^4^, identifying underlying mutational processes and providing insights into biological mechanisms. The tumor samples from each patient were assessed for microsatellite instability (MSI) using the MSIsensor-pro tool. MSI was determined by comparing these samples to normal samples from TCGA as a reference baseline. The MSI score was calculated as the percentage of somatic microsatellite sites relative to all detected microsatellite sites. Following TCGA criteria, samples with an MSI score greater than 10 were classified as MSI-H, those with a score between 4 and 10 as indeterminate, and samples with scores less than 4 as microsatellite stable (MSS). Detailed MSI scores of all samples were presented in **Table S5**.

**RNA sequencing**

Total RNA was extracted from both tissues and cultured organoids using the TRIzol Reagent (Life Technologies). RNA-seq libraries of polyadenylated RNA were prepared using the NEBNext® Ultra™ RNA Library Prep Kit for Illumina® following the manufacturer's instructions. The cDNA libraries were subsequently sequenced using the Illumina Novaseq 6000 platform, generating 150 bp paired-end reads. The raw RNA-seq reads were aligned to the human reference genome (GRCh38) using the STAR (Spliced Transcripts Alignment to a Reference) aligner. The resulting aligned reads were then quantified using RSEM, which estimated the abundance of transcripts and provided Transcripts Per Million (TPM) values for each gene. Principal Component Analysis (PCA) was conducted to visualize sample relationships based on their gene expression profiles. The pairwise Pearson correlations between samples were calculated, and the results were plotted using R package corrplot. For differential gene expression analysis, the R package EBSeq, specifically designed for single-sample RNA-Seq data, was employed. Genes showing significant differential expression were determined based on fold change and false discovery rate (FDR). Gene Ontology (GO) and Kyoto Encyclopedia of Genes and Genomes (KEGG) pathway enrichment analyses were performed using the clusterProfiler R package.

**Xenograft transplantation experiment**

NOD/ShiLtJGpt-*Prkdc*^em26Cd52^*Il2rg*^em26Cd22^/Gpt (NCG) mice, 5- to 6-week-old males, were obtained from GemPharmatech, China. All animal procedures were approved by the Animal Care Committee of Tsinghua University (the Animal Protocol 21-CYG1). To initiate the transplantation, gastric organoids were enzymatically digested into small cell clusters using TrypLE or Accutase. Approximately 3 × 10^5^ cells were then suspended in a solution of 1:1 Matrigel and complete culture medium. Subsequently, the organoid suspension was injected subcutaneously into either the left or right flank of NCG mice. After a period of two months, the grafted NCG mice were euthanized, and the xenografts were collected for subsequent histologic analysis. The length, width and weight of the subcutaneous xenografts were recorded during the analysis. The volume is calculated using the following formula: volume = (length×width^2)/2.

**Statistical analysis**

The experiments were conducted in triplicate, and statistical analysis was performed using a two-tailed Student's t-test. GraphPad Prism software was employed for all statistical analyses.

**
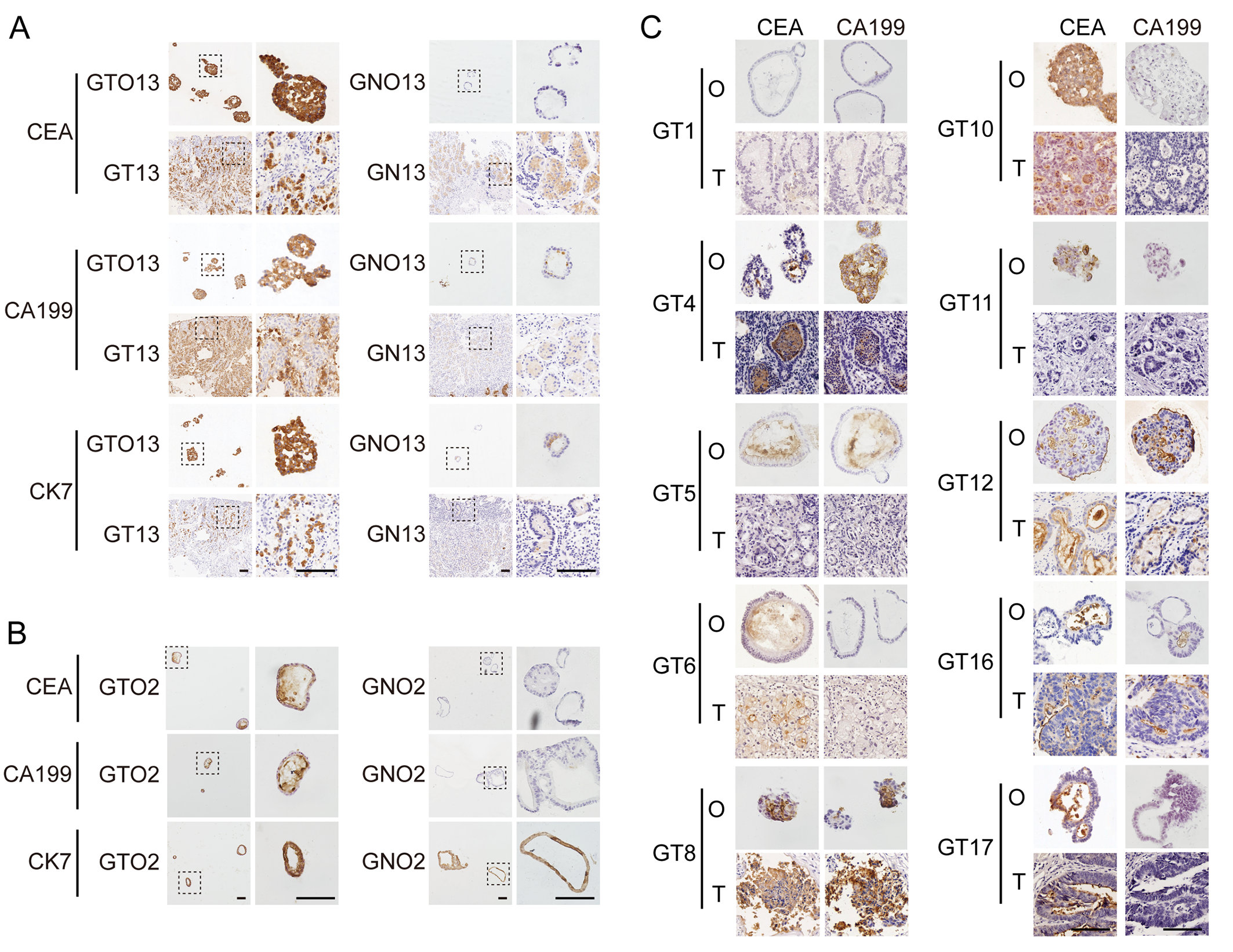
**

**Figure S1. GTOs maintain the tumor characteristics of original tumor tissues.** (**A-B**) Immunohistochemical staining of CEA, CA19-9 and CK7 in GTOs, GNOs, and the corresponding tumor and normal tissues. Scale bars: 100 μm. (**C**) Immunohistochemical staining of CEA and CA19-9 in GTOs and corresponding tumor tissues. Scale bars: 100 μm.

**
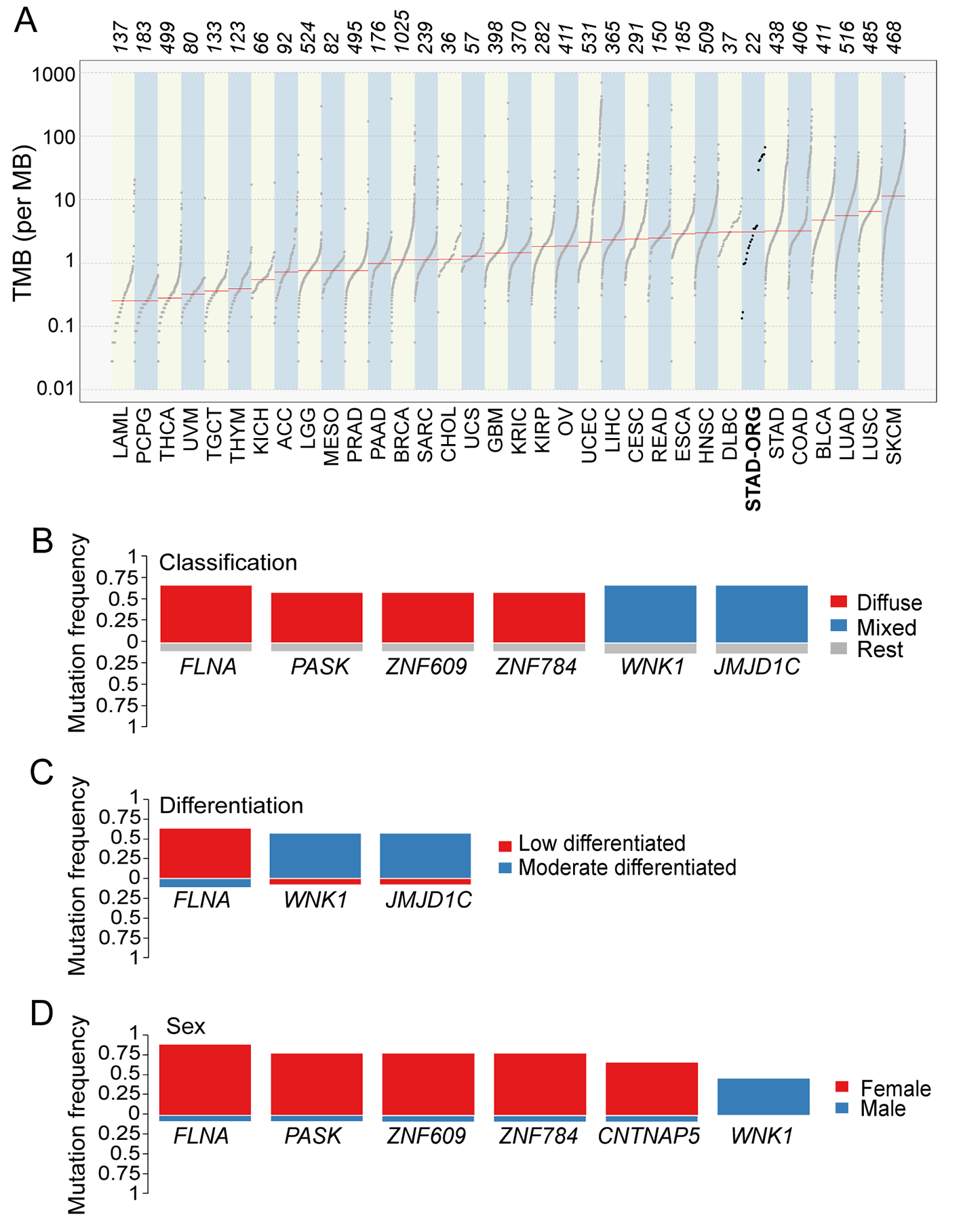
**

**Figure S2.** **Tumor characteristics in the cohort.** (**A**) Tumor mutation burden of the cohort compared to the TCGA dataset. The horizontal axis shows different cancer types, each gray dot represents a sample in the TCGA dataset, and the red horizontal lines are the median numbers of somatic mutations per megabase in the respective cancer types. Each black dot represents a sample in this study. The vertical axis represents the number of mutations per megabase. (**B-D**) Enriched mutations associated with patients’ Lauren classification (**B**), degree of tumor differentiation (**C**), and sex (**D**). The horizontal axis shows the mutated genes, and the vertical axis represents the frequency of mutated samples exhibiting specific gene mutations in each group. The rest column in grey under the diffuse group indicates the mixed and intestinal samples, while the one under the mixed group represents the diffuse and intestinal samples.


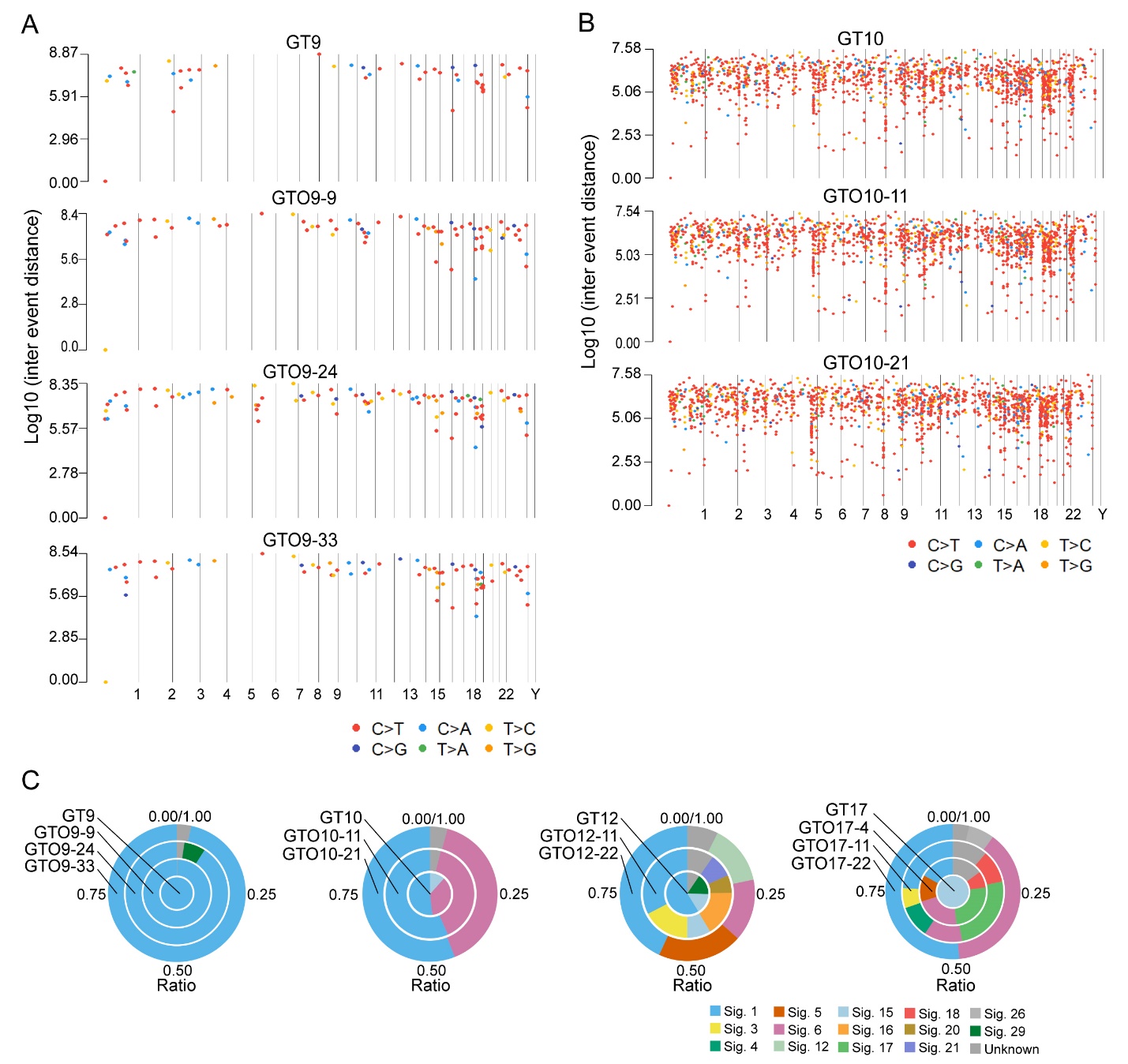


**Figure S3.** **Genomic characteristics in GTO9 and GTO10.** (**A-B**) Rainfall plots of SNVs of GTO9 (**A**) and GTO10 (**B**) at different passages and of the original tumor tissue (GT). Each dot shows a single SNV with different colors representing different substitution types. The horizontal axis represents the human genomic position and the vertical axis represents the genomic distance (log scaled) between flanking mutations in each sample. (**C**) Representative mutational signatures of GTOs and primary tumor tissues. The colors represent different signatures. Sig: signature.


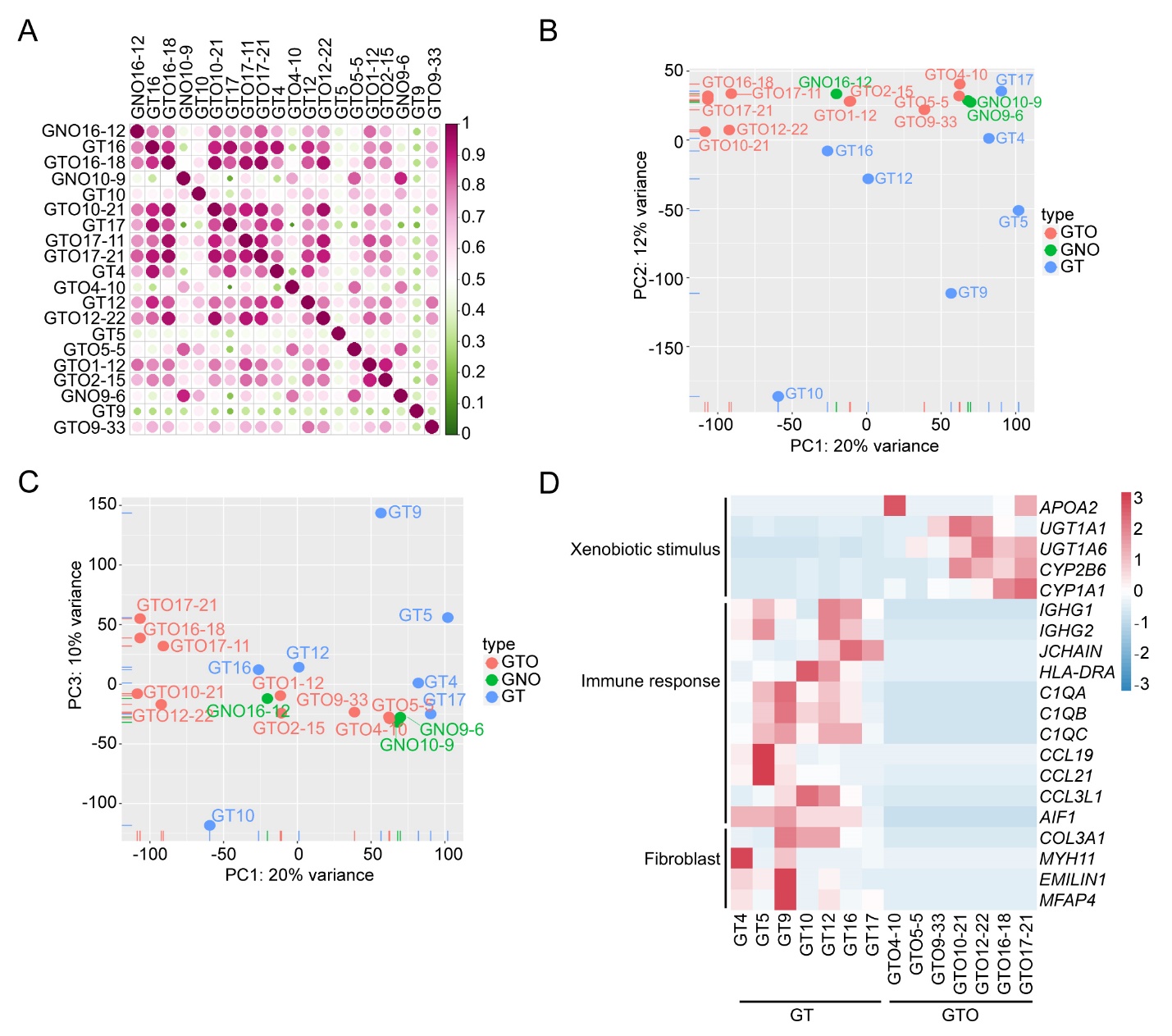


**Figure S4.** **Differentially expressed genes in gastric tumor tissues and GTOs.** (**A**) Pearson correlation of organoids and tumor tissues from different patients. (**B**) PCA plot illustrates the distribution of organoids and tumor tissues from different patients at PC1 and PC2 dimensions. (**C**) PCA plot illustrates the distribution of organoids and tumor tissues from different patients at PC1 and PC3 dimensions. The red dots represent tumor organoids, the green dots represent normal organoids, and the blue dots represent tumor tissues. PCA revealed the close proximity of different GTO subtypes, such as intestinal-type GTO1-12 with GTO2-15, diffuse-type GTO10-21 with GTO12-22, and mixed-type GTO16-18 with GTO17-11 (**B-C**). (**D**) Heatmap of differentially expressed genes in GTs compared to GTOs.


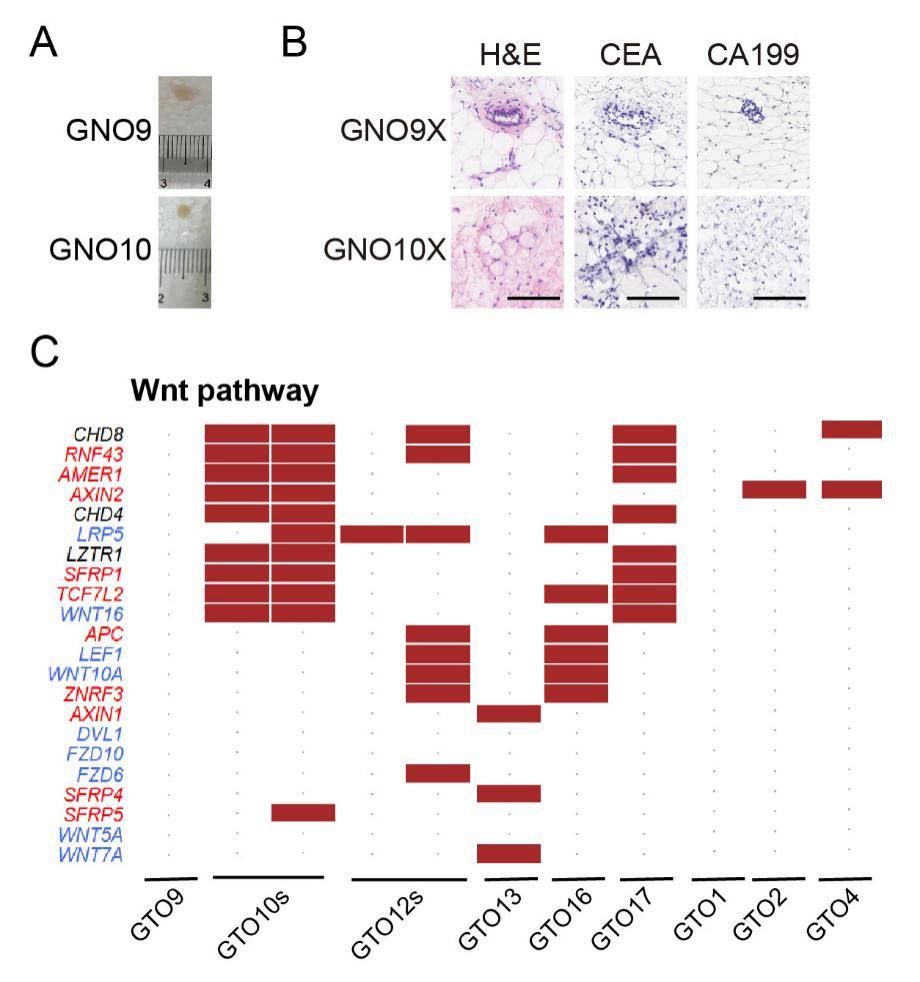


**Fi****gure S5.** **GNOs are unable to generate tumor xenografts.** (**A**) Cysts generated by GNOs in NCG-immunodeficient mice. (**B**) H&E and immunohistochemical staining of CEA and CA19-9 in the cysts formed by GNOs. Scale bars: 100 μm. (**C**) WNT pathway mutations in gastric tumor organoids. High tumorigenicity in samples with a high frequency of WNT pathway mutations (GTO10, GTO12, GTO16 and GTO17), low or absence of tumorigenicity in samples with a low frequency of WNT mutations (GTO1, GTO2, GTO4 and GTO13).


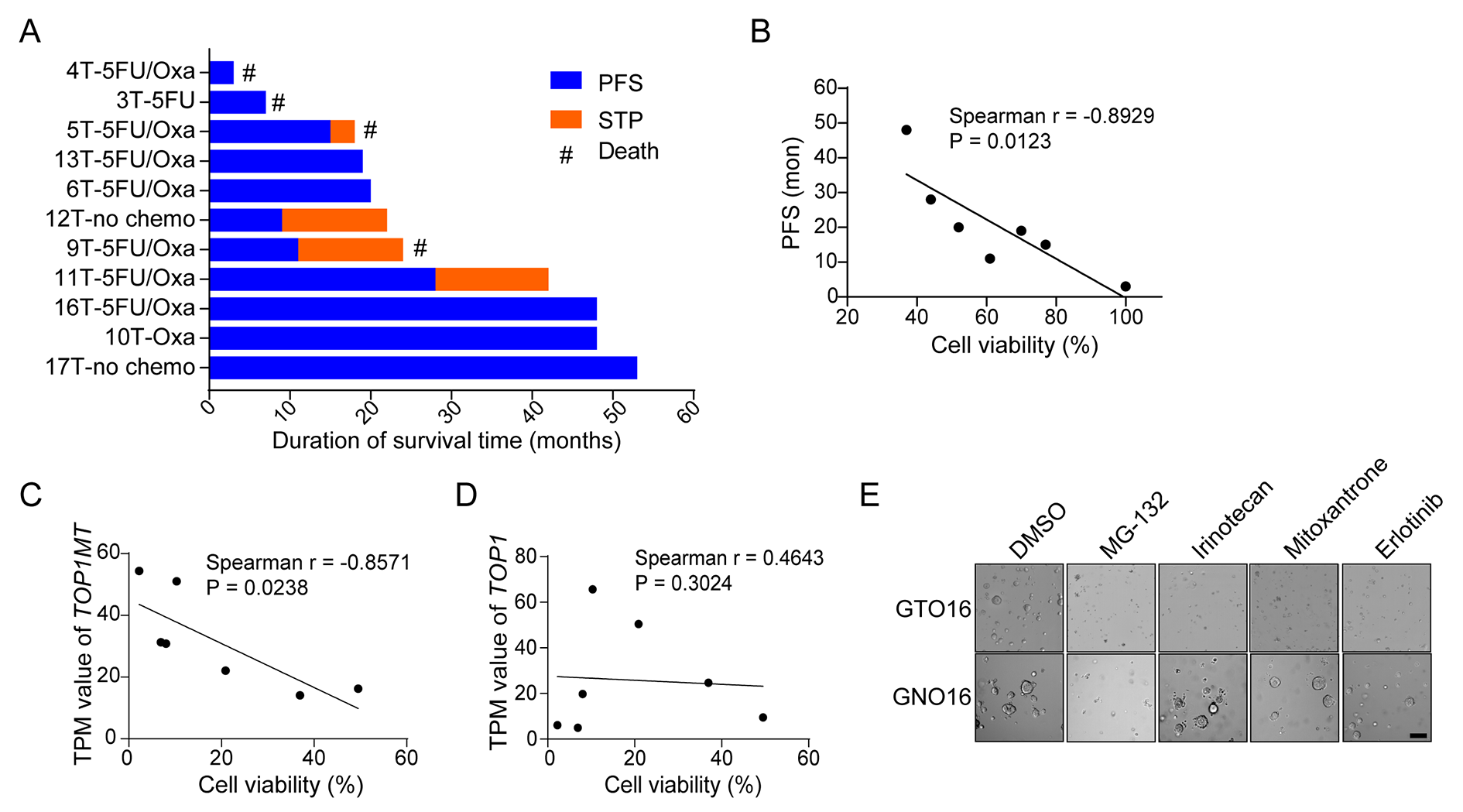


**Figure S6.** **GTOs in drug response assessment.** **(A)** Information on clinical chemotherapies, PFS time, and survival time after progression (STP) for each patient. # represents death. Oxa: oxaliplatin. **(B)** Correlation analysis between the PFS time of 7 patients (patients 4, 5, 6, 9, 10, 11, and 13) and the response of the corresponding therapeutic drugs in GTOs. **(C)** Correlation analysis between TPM value of *TOP1MT* and the cell viability after irinotecan treatment versus control group in GTOs. **(D)** Correlation analysis between TPM value of *TOP1* and the cell viability after irinotecan treatment versus control group in GTOs. Statistical analysis was performed by Spearman's correlation. (**E**) Representative images of GTO16 and GNO16 upon different drug treatments. Organoids treated with MG-132 served as the positive control. Scale bars: 100 μm.

**Supplementary Table S1**. Clinicopathological information for each patient.

**Supplementary Table S2**. Detailed levels of CEA and CA19-9 in GTOs and primary tumor tissues.

**Supplementary Table S3**. Mutations for all samples.

**Supplementary Table S4**. Critical gene mutations enriched within diverse clinicopathological patients.

**Supplementary Table S5**. MSI scores of all samples.

**Supplementary Table S6**. The composition of the signatures for each sample.

**Supplementary Table S7**. TPM values for all samples.

**Supplementary Table S8**. Steady-state plasma concentration of indicated therapeutic drugs.

**Supplementary Table S9.** Patient clinical outcomes and drug-induced cell viability in GTOs.

**References**

1 Shin, D. & Park, S. S. Clinical importance and surgical decision-making regarding proximal resection margin for gastric cancer. *World J Gastro Oncol* **5**, 4-11, doi:10.4251/wjgo.v5.i1.4 (2013).

2 Liston, D. R. & Davis, M. Clinically Relevant Concentrations of Anticancer Drugs: A Guide for Nonclinical Studies. *Clin Cancer Res* **23**, 3489-3498 (2017).

3 Schulz, M., Iwersen-Bergmann, S., Andresen, H. & Schmoldt, A. Therapeutic and toxic blood concentrations of nearly 1,000 drugs and other xenobiotics. *Crit Care* **16** (2012).

4 Alexandrov, L. B. *et al.* Signatures of mutational processes in human cancer. *Nature* **500**, 415-+, doi:10.1038/nature12477 (2013).
